# Patient-Centered Outpatient Process Optimization System Based on Intelligent Guidance in a Large Tertiary Hospital in China: Implementation Report

**DOI:** 10.2196/60219

**Published:** 2025-08-29

**Authors:** Xiaoyi Wang, Rujia Zhang, Zhihong Gao, Mengxuan Xia, Songjia Zhang, Lizheng Ge, Yuyang Zhu, Haojie Jin, Shenglian Pan, Manman Zheng, Chun Chen, Xiangyang Zhang

**Affiliations:** 1 School of Medical Humanities and Management, Wenzhou Medical University Tea Mountain Campus, Tongren Building 7B304, Ou Hai District, Wenzhou, 325035, China, 86 577 8669 9590; 2 Information Technology Section, The First Hospital of Wenzhou Medical University, Wenzhou, China; 3 Cixi Biomedical Research Institute, Wenzhou Medical University, Wenzhou, China; 4 Acupuncture and Tuina Department, Wenzhou Hospital of Traditional Chinese Medicine, Wenzhou, China; 5 Graduate School of Wenzhou Medical University, Wenzhou, China; 6 Medical Affairs Department, First Affiliated Hospital of Wenzhou Medical University, Shangcai Village, Nanbaixiang Street, Ouhai District, Wenzhou, Zhejiang, 325000, China, 0577-55578282

**Keywords:** patient-centered, intelligent system, outpatient service, waiting time, patient satisfaction

## Abstract

**Background:**

In large tertiary hospitals across China, outpatient patients often encounter the “three longs and one short” (long registration time, long waiting time, long time for medicine collection, and short time for medical treatment) phenomenon. This scenario contributes to suboptimal patient experiences and declining satisfaction with health care services. To address the issue of long waiting times, many hospitals in China have implemented a range of measures. However, these measures have only improved individual aspects of the patient experience, with limited overall impact. Currently, there is a lack of comprehensive, intelligent reform for the entire patient service process in the medical system. Therefore, there is an urgent need to integrate and optimize the entire patient service process, providing real-time intelligent guidance within hospitals. This would help reduce waiting times for patients and enhance their satisfaction.

**Objective:**

This study aims to introduce a patient-centered intelligent guidance system and report on the impact of its implementation on outpatient waiting times and patient satisfaction in hospitals.

**Methods:**

The intelligent guidance system was designed with a patient-centered approach, leveraging internet and big data technologies. The system seamlessly connects various steps of the outpatient medical process, facilitating functions including automated check-in and comprehensive intelligent guidance for patients’ medical visits, thus enhancing the efficiency and quality of health care delivery. This system has been implemented in a tertiary hospital in China. To assess the system’s effectiveness, we compared outpatient visit data, waiting time data, and patient satisfaction levels between the preimplementation and postimplementation periods from 2019 to 2022. We analyzed the changes in patients’ average waiting times and satisfaction levels after the system was implemented.

**Results:**

One year after the introduction of the intelligent guidance system, the number of outpatient visits increased from 5,067,958 to 5,456,151. The waiting time for outpatient patients was significantly reduced. The waiting time for consultation decreased by 2.84 minutes (mean 41.14, SD 2.31 min vs mean 38.30, SD 1.89 min; *P*<.001). The waiting time for examination decreased by 3.35 minutes (mean 47.83, SD 1.10 min vs mean 44.48, SD 1.67 min; *P*<.001). Consultation time increased to 3.43 minutes (mean 2.85, SD 0.03 min vs mean 3.43, SD 0.26 min; *P*<.001). After the system was launched, patient satisfaction increased from 89.99% (SD 2.78%) in 2021 to 92.72% (SD 0.18%) in 2022 (*P*=.005).

**Conclusions:**

The patient-centered intelligent guidance system reported in this study proved beneficial for tertiary medical institutions striving to alleviate the outpatient burden caused by prolonged waiting times. Through continuous transformation and upgrading of the outpatient service process centered on patients, the efficiency of outpatient services and patient satisfaction improved. Therefore, the patient-centered principle method and process integration concepts for the system can be further promoted and implemented.

## Introduction

### Context

With the continuous global development of the economy and society, people have developed higher expectations of their health systems. The World Health Organization (WHO) has proposed that the current health system is a physician-driven system overly focused on disease, neglecting the patient experience, such that nearly half of patients are dissatisfied with the system [1]. Therefore, the WHO proposed the principle of people-centeredness, which aims to create a patient-centered health system. Many countries have actively responded to this principle, including Peru [2], Canada [3], and Germany [4]. Several scholars have conducted research on integrating patient-centered principles into the nursing field [5,6]. Researchers have experimentally compared inpatient care experiences between systems implementing and not implementing patient-centered principles, demonstrating improved service quality and optimized patient experiences [7].

### Problem Statement

Patient-centered principles remain limited in China due to its vast population and relatively scarce high-quality medical resources. Additionally, China’s medical process differs from other nations where strict family doctor first-visit systems manage common illnesses locally and refer complex cases to hospitals [8]. China’s hospitals are graded by the government (Levels I, II, and III) [9]. Level I and II hospitals manage common illnesses, while Level III (tertiary) hospitals, equipped with the best resources, handle critical cases [10,11]. Chinese patients choose their hospitals by themselves [12]. Consequently, many Chinese patients seek care for common ailments like colds at large tertiary hospitals, compelling these institutions to handle both minor and serious conditions [13]. This leads to persistent overcrowding and queues.

**Figure 1.**
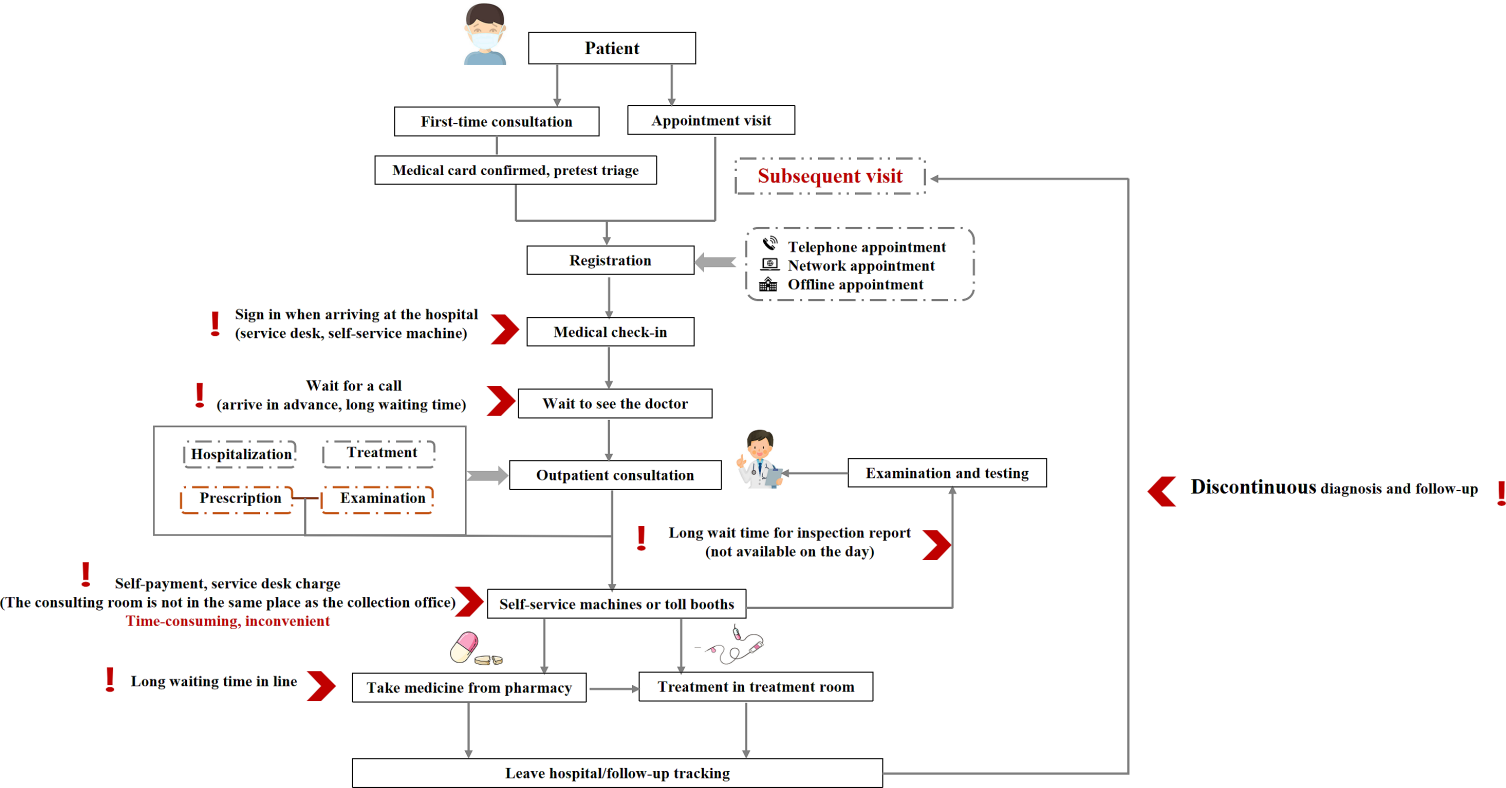
Traditional outpatient procedures and existing problems.

During observation of a traditional hospital consultation process, most first-time patients exhibited confusion upon arrival (Figure 1). They struggled to locate key departments like consultation rooms or pharmacies, leading to repeated queuing at information desks for staff guidance. This manual consultation dependency significantly prolongs waiting times and wastes patient time. Doctors’ consultation times for each patient receiving medical services are shortened accordingly. This leads to a situation in which patients need a long time to register, queue for long periods, take a long time for examinations, and see a doctor for a short amount of time [14]. Excessive waiting times also degrade the patient’s experience of care, resulting in decreased satisfaction [15-17].

### Similar Interventions

Prolonged outpatient waiting times have been found to lower patient satisfaction across multiple countries, including China, Canada, Malaysia, and the United States [18-20]. Studies have examined patient-centered care principles in China. Surveys assessed patients’ perceptions and satisfaction [21], while research explored the application of patient-centered care principles to health care challenges [22]. Analysis of the 2015 China National Patient Survey found these principles improve care quality, efficiency, and satisfaction [23]. Many public tertiary hospitals in China have made numerous attempts at improvement [13]. For example, a public tertiary hospital in Southern China balanced supply-demand interventions by simplifying appointments, regulating doctor attendance, and implementing app-based visit reminders [14]. Postintervention, this hospital reduced patient waiting time by 28.3 minutes (from 179.7 to 151.4 min), while satisfaction scores rose from 89.10 to 90.26 [14]. Other hospitals have optimized outpatient processes by introducing current technologies, such as web-based approaches to health care delivery [24]. Other researchers have designed smart navigation systems to shorten queues [25].

Previous health care reforms typically addressed fragmentary treatment aspects while neglecting overall care pathways and patient habits. For example, underutilized artificial intelligence guidance systems and app-based solutions often exclude older adult patients due to poor accessibility [26]. These fragmented initiatives fail to address the widespread issue of “three long and one short” (long registration, waiting, and payment times, coupled with short consultation times). Although long-term solutions such as equalizing access to high-quality medical resources and implementing a first-visit system are essential, they lack immediate impact. In the short term, optimizing outpatient processes in tertiary hospitals offers a practical approach to reducing wait times and enhancing patient experience.

**Figure 2.**
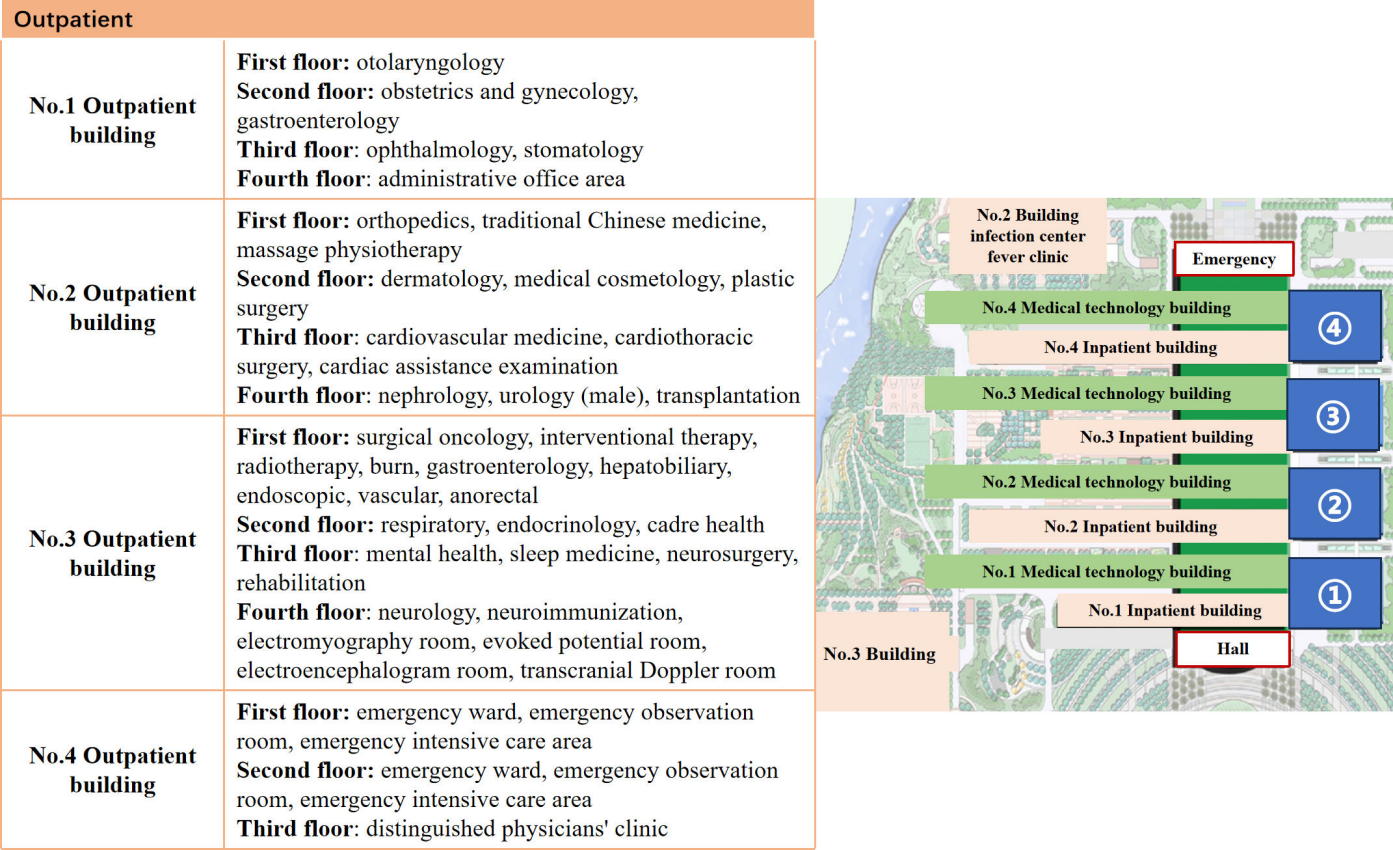
Hospital map and department distribution.

This report examines the implementation of a patient-centered intelligent guidance system at a large Chinese tertiary hospital (Figure 2 shows its outpatient building and department layout). The report adheres to the iCHECK-DH (Guidelines and Checklist for the Reporting on Digital Health Implementations) (Checklist 1) [27].

## Methods

### Aims and Objectives

This study evaluated the intelligent guidance system’s efficacy in reducing treatment waiting times and improving patient satisfaction. Herein, we discuss the system’s development and implementation, then compare waiting time and satisfaction data preimplementation and postimplementation.

### Blueprint Summary

**Figure 3.**
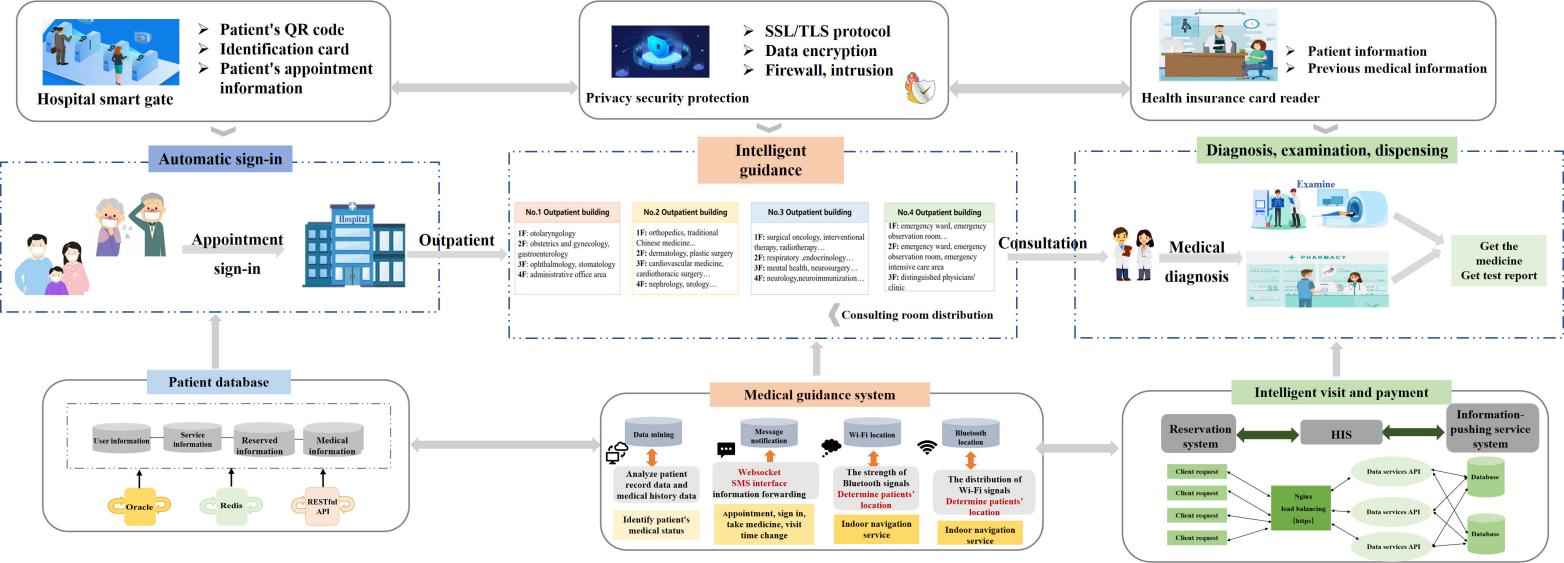
Upgraded intelligent guidance system.

The intelligent guidance system, grounded in patient-centered principles, integrates multisource data and smart sensing technologies to streamline the entire treatment process. It features an automatic check-in module and indoor navigation system linked to hospital databases and installed at entrance gates (Figure 3). Using identity recognition for contactless check-in and hybrid Wi-Fi/Bluetooth positioning for real-time tracking, the system analyzes treatment data to deliver personalized navigation routes via SMS text messaging notifications. It dynamically adjusts guidance based on real-time positioning and treatment progress, ensuring seamless transitions across consultations, examinations, pharmacy visits, and follow-ups until patient departure. The intelligent medical guidance system avoids mandatory app downloads, using SMS text messaging for inclusive notifications [25]. Unlike traditional systems requiring manual input [26,28], it integrates registration, check-in, and examinations, providing real-time updates and automatic route planning (Table 1). It acts as a personal medical assistant, guiding patients throughout their visit.

**Table 1.** Comparison between traditional guidance systems and intelligent guidance systems.

Category	Traditional guidance systems	Intelligent guidance system
Service acquisition	Download the hospital app or use a specialized device	A phone that can receive text messages
Main function	Simplified functionality: dedicated exclusively to hospital navigation services	Diverse functionalities: assist patients with automatic check-in, link medication pickup, examinations, and treatments, dynamically update patients’ medical information in real time, and automatically plan routes to guide them
Features	Noncontinuous service	Continuous service
Principle	Non–patient-centered	Patient-centered

### Technical Support for the Intelligent Guidance System

The upgraded system uses a cloud-native microservice system architecture that is convenient for deployment in different environments. Each system is independent and provides better isolation, scalability, ease of maintenance, and other characteristics. The main technology stacks used by the software are Spring Cloud, VUE, Rabbit MQ message queuing, Redis caching, distributed scheduling tasks, Docker, Kubernetes, and DevOps. The cloud-native technology architecture can be used to quickly expand service resources and perform peak business data processing in hospitals. The daily throughput can reach tens of millions, the response time for most requests is in milliseconds, and the data processing capacity can rapidly expand with the amount of data.

### Target

The intelligent guidance system was launched in October 2020 at a large tertiary hospital in China and aimed to reduce excessive outpatient waiting times and enhance patient satisfaction. The system provides services to all outpatient patients annually at the hospital. After two years of operation, it has demonstrated initial effectiveness, with subsequent adoption by other tertiary hospitals across China.

### Ethical Considerations

This retrospective analysis of fully anonymized hospital operational data received ethical approval from the Ethics Committee of Wenzhou Medical University (approval number 2020‐075), which explicitly authorized reuse of existing system data without additional consent. Patients retained an opt-out mechanism by rejecting the system’s terms through the initial pop-up notification. We are committed to strict confidentiality regarding the personal information and privacy of all research participants, and we have implemented multiple measures to ensure data privacy and security. The collected health and personal data will be stored using industry-standard encryption techniques, and only authorized researchers will have access to these data. All data usage and storage processes will follow strict data management policies to prevent unauthorized access and data breaches. No compensation was provided due to the noninterventional nature of this research using anonymized data.

### Data

The intelligent guidance system retrieves real-time patient data, including appointments and Wi-Fi– and Bluetooth-based locations, via the health information system (HIS) interface, excluding sensitive information. It analyzes patient locations and schedules, generating optimized navigation routes and updating queuing systems upon check-in. Data are securely stored in encrypted local servers and synchronized with the HIS every 10 seconds; anonymized trajectory data are retained for system optimization, while original logs are purged quarterly. Patients access medical records via the hospital portal after ethical approval and informed consent, with security ensured through localized storage, role-based access control, 2-factor authentication, and intranet-restricted analysis. The system interfaces with the HIS, electronic medical record platform, and Internet of Things devices via application programming interface synchronization, enabling real-time updates and anonymized navigation logs for optimization, complying with China’s Personal Information Protection Law (Article 28). Patients authorize data usage through electronic agreements, with privacy-by-design principles, encrypted transmission, and strict adherence to data localization (Cybersecurity Law, Article 37) and classified protection (Data Security Law, Article 21).

### Interoperability

The intelligent guidance system achieves real-time integration with the hospital’s HIS system, synchronizing patient registration and medical information while automatically aligning diagnostic data with optimal treatment pathways. The system supports cross-brand communication devices and navigation terminals, ensuring interoperability across various patient devices within the hospital, thereby enhancing the convenience and accuracy of navigation routes.

### Participating Entities

The intelligent guidance system was developed by the IT department of a public hospital. Guided by government policies emphasizing patient service optimization, the hospital’s IT center manages HIS integration, enabling secure real-time data exchange across registration, testing, and pharmacy services, along with system maintenance. The outpatient department contributes to route planning and status updates, while the logistics department handles device deployment. Clinical staff provide practical feedback for system improvement, with funding primarily from hospital resources.

### Budget Planning

The intelligent guidance system underwent a 12-month development and 3-month pilot deployment. Budget allocation prioritized software and algorithm optimization (50%), covering navigation engine development, HIS integration, and patient interface design. Hardware integration, including Bluetooth beacons and signage, accounted for 20%. Cross-department collaboration received 15%, change management for process updates 10%, and the remaining 5% supported stress testing and patient flow optimization during the pilot phase.

### Sustainability

The intelligent guidance system achieves routine operation through integration with the HIS. Financially, initial development was funded by the hospital’s IT budget, with sustainable revenue growth realized through enhanced efficiency and increased patient volume. Operationally, the system reduces the need for manual guidance staff, saving human resources while optimizing patient flow and reducing in-hospital stay duration.

## Implementation (Results)

### Coverage

We used 2019 as the baseline to evaluate waiting times before the system’s October 2020 launch, excluding 2020 due to COVID-19 impacts and system buffer time. Data from 2019, 2021, and 2022 were analyzed for waiting time improvements, while 2021‐2022 data tracked satisfaction trends. Patient satisfaction was measured using structured questionnaires with 5-point Likert scales. Respondents were patients who received treatment at the hospital. After completing all medical consultation procedures, satisfaction questionnaires were automatically pushed by the system. Consultation and examination waiting times for patients aged ≥60 were analyzed separately, though satisfaction data, collected anonymously, lacked age stratification.

The changes in the basic situation of outpatients before and after system optimization and upgrade are summarized in Table 2, which includes the total number of outpatients in 2019, 2020, 2021, and 2022, the sex distribution of outpatients, the age distribution, and the number of examination bills issued in recent years. From 2019 to 2022, the total number of outpatient visits fluctuated. The number of visits increased from 5,067,958 in 2019 to 5,456,151 in 2022. The growth rate during this period was 7.66%. We categorized the patients into the following groups: younger individuals aged 18 years and under, adults aged 19‐40 years, adults aged 41‐59 years, and older adults aged ≥60 years. From 2019 to 2022, the number of outpatients in different age groups steadily increased. Patients aged 41‐59 years accounted for the highest proportion of all outpatients across all study years: 38.6% in 2019, 39.5% in 2020, 40.0% in 2021, 39.5% in 2022. Patients aged 18 accounted for the lowest proportion. The proportion of female outpatients in recent years was generally greater than that of male outpatients. Except for 2020, the proportion of male patients (56%) was higher than the proportion of female patients (44%). The number of invoices also showed an increasing trend from 3,555,896 in 2019 to 3,778,609 in 2022, with a growth rate of 6.26%.

**Table 2.** Characteristics of hospital outpatients from 2019 to 2022.

Characteristics	2019	2020	2021	2022
Total patients	5,067,958	4,327,575	5,160,841	5,456,151
Number of invoices	3,555,896	3,075,948	3,665,579	3,778,609
Age group (years), n (%）				
≤18	302,925 (6)	220,846 (5.1)	264,557 (5.1)	277,774 (5.4)
19‐40	1,563,163 (30.8)	1,283,780 (29.7)	1,538,196 (29.8)	1,542,894 (30)
41‐59	1,956,001 (38.6)	1,710,187 (39.5)	2,062,394 (40)	2,029,534 (39.5)
≥60	1,245,869 (24.6)	1,112,762 (25.7)	1,295,694 (25.1)	1,292,841 (25.1)
Gender, n (%）				
Male	2,238,480 (44.2)	3,075,948 (56)	2,254,364 (43.7)	2,218,642 (43.9)
Female	2,829,478 (55.8)	2,415,691 (44)	2,906,477 (56.3)	2,829,622 (56.1)

### Outcomes

**Figure 4.**
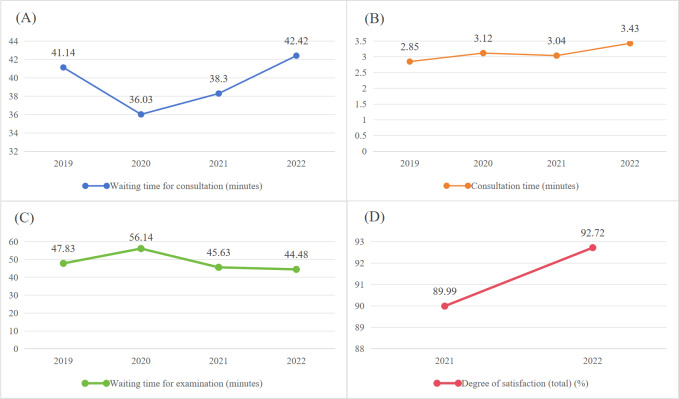
Changes in total patient wait times and satisfaction from 2019 to 2022. (A) Waiting time for consultation. (B) Consultation time. (C) Waiting time for examination. (D) Degree of satisfaction (total).

Patient waiting time includes consultation and examination waits. Consultation time is the interval between check-in and doctor login; examination time is the interval between prescription and formal check-in. From 2019 to 2021, overall waiting times decreased: consultation wait times dropped by 3.39 minutes (41.14 to 38.30 min) and examination wait times dropped by 3.35 minutes (47.83 to 44.48 min), while consultation duration increased from 2.85 to 3.43 minutes (Table 3). For patients aged ≥60 years, wait times decreased by 1.82 minutes (42.37 to 40.55 min) and examination wait times decreased by 4.41 minutes (52.04 to 47.63 min), with consultation duration rising from 3.14 to 3.40 minutes (Table 4). Figures4  5 visualize these trends. After optimization and upgrading of the system, patients’ satisfaction with the outpatient process continued to improve, and overall satisfaction increased from 89.99 in 2021 to 92.72 in 2022 (*P*=.005). Figure 4 more clearly visualizes the change in waiting time and the change in satisfaction.

**Table 3.** Total patient wait times and satisfaction from 2019 to 2022.

Study phase	2019, mean (SD)	2020, mean (SD)	2021, mean (SD)	2022, mean (SD)	*F* test (*df*)	*P* value
Waiting time for consultation (minutes）	41.14 (2.31)	36.03 (2.78)	38.30 (1.89)	42.42 (2.31)	17.974 (3)	<.001
Waiting time for examination (minutes）	47.83 (1.10)	56.14 (13.34)	45.63 (1.67)	44.48 (1.56)	7.219 (3)	<.001
Consultation time	2.85 (0.03)	3.12 (0.31)	3.04 (0.96)	3.43 (0.26)	16.198 (3）	<.001
Degree of satisfaction (total）	—^a^	—	89.99 (2.78)	92.72 (0.18)	9.981 (11.09)^b^	.005

a Not applicable.

b Degrees of freedom are presented as decimals due to the use of Welch ANOVA correction for violation of the homogeneity of variances assumption (Levene test, *P*<.05). Standard ANOVA results are reported when assumptions were met.

**Table 4.** Waiting times for patients aged ≥60 years from 2019 to 2022.

Study phase	2019, mean (SD)	2020, mean (SD)	2021, mean (SD)	2022, mean (SD)	*F* test (*df*)	*P* value
Waiting time for consultation (minutes）	42.37 (2.62)	37.61 (4.02)	40.55 (1.80)	43.68 (2.54)	10.16 (3)	<.001
Waiting time for examination (minutes）	52.04 (1.52)	65.30 (21.25)	47.63 (1.60)	46.58 (1.54)	7.75 (3)	<.001
Consultation time	3.14 (0.04)	3.47 (0.33)	3.40 (0.10)	3.72 (0.22)	16.35 (3)	<.001

**Figure 5.**
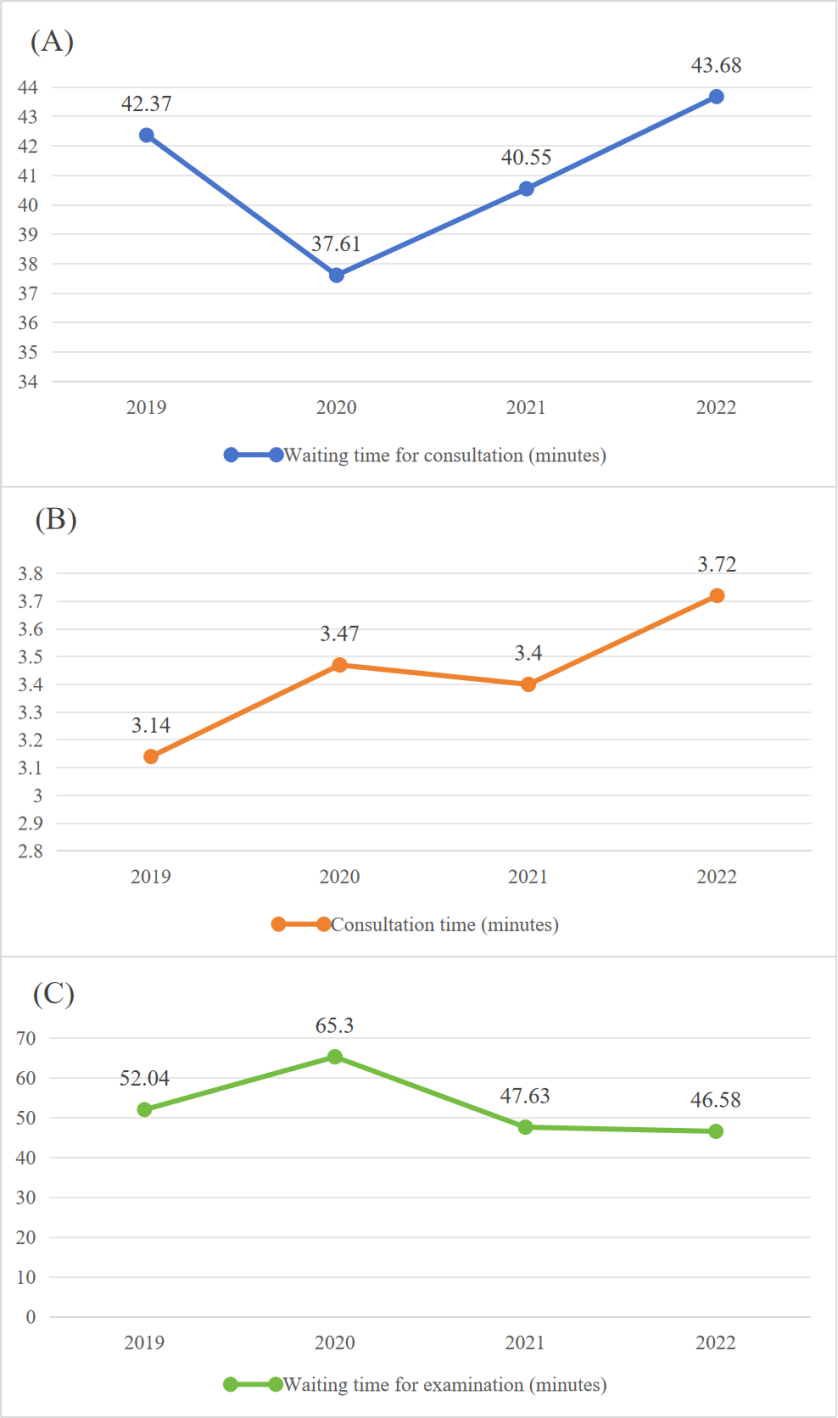
Changes in older patient wait times and satisfaction from 2019 to 2022. (A) Waiting time for consultation. (B) Consultation time. (C) Waiting time for examination.

## Discussion

### Principal Findings

Applying patient-centered principles, our study transformed a tertiary hospital’s medical service process through an intelligent guidance system. This mobile-guided system integrates admission, waiting, and examinations, significantly reducing queues and exam waits while extending consultation times and improving outpatient satisfaction.

Reduced patient waiting times, this reform’s primary outcome, align with multiple intervention studies. South Korean physicians used queuing theory to achieve a 30% reduction in outpatient waiting times [20]. A New York pediatric clinic reduced waiting times by 3.2-8.8 minutes through targeted interventions [29]. In China, a tertiary hospital streamlined nonemergency registrations, cutting waiting times from 25.05 to 1.00 minutes [13]. Another redesigned its outpatient pharmacy, halving prescription pickup times from 1 hour to 30 minutes [30]. Similarly, a hospital implementing artificial intelligence–assisted consultations reduced median waiting times from 1.97 to 0.38 hours [31]. However, the waiting time for visits in 2022 increased by 1.28 minutes compared to 2019. The system initially went live in 2020, and the time was reduced in 2021. Considering the huge demand for high-quality medical resources and support for system optimization, the hospital opened more appointments in the same time frame. In the future, after further improvement of the system, it is hoped that the number of tertiary hospitals included will continue to expand in stages to meet the medical needs of more patients.

Another significant benefit of implementing the intelligent guidance system was the increase in the duration of patient consultation time. Consultation time is a crucial factor as it influences the quality of care and patient satisfaction [32-34]. Notably, extending consultation time may enhance satisfaction more substantially than reducing waiting time [9]. Consultation time in Sweden is 10‐20 minutes [35]. Research conducted in the United States indicated that the average consultation time ranges from 10 to 15 minutes [36]. At a tertiary hospital in China, the average consultation time in 2017 was 6.52 minutes. Globally, consultation durations remain consistently shorter than patient waiting times despite cross-country variations. One hospital even reported reduced consultation times postreform, decreasing from 6.52 to 3.12 minutes [14]. Our smart guidance system progressively extended consultation time. This improvement stemmed from streamlined registration and navigation processes, which reduced missed appointments and clinic crowding, freeing physicians to concentrate on clinical consultations rather than on maintaining order.

Patient satisfaction serves as a crucial gauge of health care quality [37]. A study conducted in Canada demonstrated a significant association between waiting time and patient satisfaction, reporting an odds ratio of 0.92 (95% CI 0.86‐0.98; *P*=.01) [38]. Nonphysician tasks like registration and pharmacy services prolong wait times, thus reducing patient satisfaction given the inverse wait time/satisfaction relationship [17,39,40]. Our research findings are consistent with this. Tertiary hospitals tend to occupy large areas and have many campuses and buildings. Even when landmarks and walls indicate their locations, patients often get lost in hospitals [41]. The intelligent navigation system addresses this by guiding patients to their appointments, embodying patient-centered care. It significantly boosts satisfaction.

This study demonstrates the significant effectiveness of applying an intelligent guidance system to optimize patient flow in a tertiary hospital, with this experience holding clear generalizability for other tertiary hospitals facing similar challenges such as high patient volume, complex spatial layouts, and prolonged waiting times. The system’s core logic—integrating admission check-in, treatment waiting, and examination scheduling with precise mobile guidance—combined with its modular design, can be directly implemented in other large tertiary hospitals to efficiently optimize patient visit processes. Sustainability relies on routine maintenance rather than continuous technical investment, while reduced waiting times increase throughput, allowing more appointments within existing time frames to boost revenue. For successful adoption, hospitals must preserve traditional channels for older adult patients, implement phased pilots starting with high-volume departments, and thoroughly evaluate local contexts including digital capabilities, regional disparities, and patient demographics before staged adaptation.

### Limitations and Lessons Learned

The implementation phase consistently presented the greatest challenges, aligning with common industry observations. First, indoor positioning inaccuracies occasionally caused patient navigation errors, requiring supplementary staff guidance that diminished expected efficiency gains. Second, challenges in reaching all patient groups remained, particularly older adults. Despite including features like SMS text message reminders to help bridge the gap, a significant “digital divide”persisted. Most older adult patients preferred traditional methods like physical queuing or in-person assistance. This lower adoption rate among older adults meant the system could not deliver its intended benefits equally to everyone. Future work will tailor research and designs specifically for older adult patient adoption. Finally, deep integration with complex hospital systems caused intermittent interface instability and data synchronization delays, impacting workflow accuracy. During high patient volumes, peak-hour response delays exposed deficiencies in initial stress testing and capacity planning.

Nevertheless, methodological limitations warrant caution. Unadjusted confounders such as departmental variations may partially explain outcomes. For example, the Obstetrics, Surgery, and Cardiology departments face longer waits due to complex workflows. Visit type differences also exist, where initial consultations require more time than follow-ups. Temporal factors like peak-hour or weekday clinics with higher volumes further complicate analysis. Satisfaction surveys incurred self-selection bias as anonymously distributed mobile questionnaires prevented response rate tracking, restricting analysis to voluntary respondents who systematically differ from nonrespondents. Although age, sex data, and wait times for those aged ≥60 years were documented, key potential confounders including education level, digital literacy, and visit type lacked statistical control. Consequently, outcome associations may be confounded despite standardized HIS metrics, hindering the exploration of variables influencing system usage. Future studies need stratified randomization, multivariable adjustment, and enhanced response strategies to isolate intervention effects.

### Conclusions

The WHO advocates patient-centered care to improve medical experiences. Implementing intelligent guidance systems alleviates outpatient issues like prolonged queuing, excessive waits, and low satisfaction through continuous service. This model offers global reference value, particularly for China and similar settings, by upgrading processes with intelligent technologies to reduce waits and increase satisfaction.

## Supplementary material

10.2196/60219 Checklist 1 iCHECK-DH checklist.

## Abbreviations

HIS: health information system
iCHECK-DH: Guidelines and Checklist for the Reporting on Digital Health Implementations
WHO: World Health Organization
