# Supplementary material for: Patient-Centered Outpatient Process Optimization System Based on Intelligent Guidance in a Large Tertiary Hospital in China: Implementation Report
Source: JMIR Med Inform. 2025 Aug 29;13:e60219. doi: 10.2196/60219 (PMC12396732; doi:10.2196/60219)
Supplement: Checklist 1 [file medinform-v13-e60219-s001.docx]

Checklist of iCHECK-DH guidelines. iCHECK-DH: Guidelines and Checklist for the Reporting on Digital Health Implementations.

| **section** |  | **Item** | **Description** |
| --- | --- | --- | --- |
| **Title** | 1 | Title  (M^[[1]](#footnote-0)^) | A Patient-centered Outpatient Process Optimization System Based on Intelligent Guidance in a Large Tertiary Hospital in China：Implementation Report |
| **Abstract** | 2 | Abstract  (M) | See in Abstract. The patient-centered intelligent guidance system has reduced outpatient waiting times and improved patient satisfaction by optimizing the patient service process. |
| **Introduction** | 3 | Context  (M) | See in Introduction section. This section primarily elaborates on the patient - centered principle. In contemporary medical practice, the healthcare process places greater emphasis on optimizing the patient - centered experience, aiming to enhance every aspect of patients' encounters during medical consultations and treatments. |
|  | 4 | Problem statement  (M) | See in Introduction section. In current China, there exists a problem of long waiting times in tertiary - level hospitals. This not only brings patients a poor medical experience but also leads to a decline in patient satisfaction. |
|  | 5 | Similar Interventions  (M) | See in Introduction section. Numerous hospitals have been endeavoring to implement reforms through diverse approaches. However, the problem of excessively long patient waiting times persists. |
| **Methods** | 6 | Aims and Objectives  (M) | See in Methods section. In this study, our objective was to evaluate the efficacy of the intelligent guidance system in reducing patients’ waiting times for medical treatment and enhancing patient satisfaction. First, we introduced the development and implementation the intelligent guidance system. Subsequently, we collected and compared data on waiting times and patient satisfaction before and after the system began to operate. |
|  | 7 | Blueprint summary  (M) | See in Methods section. The Intelligent Guidance System, grounded in patient-centered principles, integrates multi-source data and smart sensing technologies to streamline the entire treatment process. It features an automatic check-in module and indoor navigation system, linked to hospital databases and installed at entrance gates |
|  | 8 | Technical Design  (M) | See in Methods section. The upgraded system, featuring a cloud - native microservice architecture, is easy to deploy in various environments. Each independent system offers better isolation, scalability, and maintainability. Its main tech stacks include Spring Cloud, VUE, Rabbit MQ, Redis, distributed scheduling, Docker, Kubernetes, and DevOps. This architecture enables rapid service resource expansion and peak - time hospital data processing. With a daily throughput of tens of millions, most requests respond in milliseconds, and data processing capacity grows with data volume. |
|  | 9 | Target  (M) | See in Methods section. The Intelligent Guidance System was launched in October 2020 at a large tertiary hospital in China, aimed at reducing excessive outpatient waiting times and enhancing patient satisfaction. The system provides services to all outpatient patients annually at the hospital. After two years of operation, it has demonstrated initial effectiveness, with subsequent adoption by other tertiary hospitals across China. |
|  | 10 | Data  (M) | See in Methods section. |
|  | 11 | Interoperability  (M) | See in Methods section.The Intelligent Guidance System achieves real-time integration with the hospital's HIS system, synchronizing patient registration and medical information, while automatically aligning diagnostic data with optimal treatment pathways. The system supports cross-brand communication devices and navigation terminals, ensuring interoperability across various patient devices within the hospital, thereby enhancing the convenience and accuracy of navigation routes. |
|  | 12 | Participating entities  (M) | See in Methods section. The Intelligent Guidance System was developed by the IT department of a public hospital. Guided by government policies emphasizing patient service optimization, the hospital's IT center manages HIS integration, enabling secure real-time data exchange across registration, testing, and pharmacy services, along with system maintenance. The outpatient department contributes to route planning and status updates, while the logistics department handles device deployment. Clinical staff provide practical feedback for system improvement, with funding primarily from hospital resources. |
|  | 13 | Budget Planning  (M) | See in Methods section. The Intelligent Guidance System underwent a 12-month development and 3-month pilot deployment. Budget allocation prioritized software and algorithm optimization (50%), covering navigation engine development, HIS integration, and patient interface design. Hardware integration, including Bluetooth beacons and signage, accounted for 20%. Cross-department collaboration received 15%, change management for process updates 10%, and the remaining 5% supported stress testing and patient flow optimization during the pilot phase. |
|  | 14 | Sustainability  (M) | See in Methods section. The Intelligent Guidance System achieves routine operation through integration with the Hospital Information System (HIS). Financially, initial development was funded by the hospital's IT budget, with sustainable revenue growth realized through enhanced efficiency and increased patient volume. Environmentally, the system reduces paper consumption and decreases energy usage by optimizing patient flow and reducing in-hospital stay duration. |
| **RESULTS** | 15 | Coverage  (M) | See in Results section. |
|  | 16 | Outcomes  (M) | See in Results section. |
|  |  | Lessons learned  (M) | See in Discussion section (Principal Findings, and Limitations). |
|  | 17 |  |  |
|  | 18 | Unintended consequences  (NM^[[2]](#footnote-1)^) | NA |
| **Discussion** | 19 | Conclusion  (M) | See in Conclusion section. |
| **General** | 20 | General  (NM) | NA |

1. M: Mandatory item [↑](#footnote-ref-0)
2. NM : Non-mandatory item [↑](#footnote-ref-1)
